# Hsp12.6 Expression Is Inducible by Host Immunity in Adult Worms of the Parasitic Nematode *Nippostrongylus brasiliensis*

**DOI:** 10.1371/journal.pone.0018141

**Published:** 2011-03-23

**Authors:** Naoki Arizono, Minoru Yamada, Tatsuya Tegoshi, Yutaka Takaoka, Mika Ohta, Toshiyuki Sakaeda

**Affiliations:** 1 Department of Medical Zoology, Kyoto Prefectural University of Medicine, Kyoto, Japan; 2 Division of Applied Genome Science and Bioinformatics, Kobe University Graduate School of Medicine, Kobe, Japan; 3 Center for Integrative Education of Pharmacy Frontier (Frontier Education Center), Graduate School of Pharmaceutical Sciences, Kyoto University, Kyoto, Japan; Universidade Federal de Minas Gerais, Brazil

## Abstract

Heat shock proteins (Hsp) are a family of stress-inducible molecular chaperones that play multiple roles in a wide variety of animals. However, the roles of Hsps in parasitic nematodes remain largely unknown. To elucidate the roles of Hsps in the survival and longevity of nematodes, particularly at the 2 most critical stages in their lifecycle, the infective-L3 stage and adult stage, which is subjected to host-derived immunological pressure, we examined the temporal gene transcription patterns of Hsp12.6, Hsp20, Hsp70, and Hsp90 throughout the developmental course of the nematode *Nippostrongylus brasiliensis* by reverse transcriptase real-time PCR. *Nb*-Hsp70 and *Nb*-Hsp90 expression were observed throughout the nematode's lifecycle, while the expression of *Nb*-Hsp20 was restricted to adults. Interestingly, *Nb*-Hsp12.6 showed a biphasic temporal expression pattern; i.e., it was expressed in infective-L3 larvae and in adults during worm expulsion from immunocompetent rats. However, the activation of *Nb*-Hsp12.6 in adult worms was aborted when they infected permissive athymic-*rnu/rnu* rats and was only marginal when they infected mast-cell-deficient *Ws/Ws* rats, which exhibited a low response of rat mast cell protease (RMCP) II and resistin-like molecule (Relm)- β expression compared to those observed in immunocompetent rats. Moreover, the activation of *Nb*-Hsp12.6 was reversed when adult worms were transplanted into the naive rat intestine. These features of *Nb*-Hsp12.6, the expression of which is not only stage-specific in infective-L3, but is also inducible by mucosal immunity in adults, have implications for the survival strategies of parasitic nematodes in deleterious environmental conditions both outside and inside the host.

## Introduction

Worldwide, hundreds of millions of people are afflicted by infection with intestinal nematodes, such as hookworms and *Strongyloides* spp. For successful infection and lifecycle continuation, these soil-transmitted nematodes must survive in two different environments: outside and inside the host. The first critical stage for survival is the infective-L3 stage, which must live in a harsh environment until they find and successfully infect an appropriate host. It has been suggested that infective-L3 larvae are analogous to dauer (L3) larvae, the dormant survival stage of the free-living nematode *Caenorhabditis elegans*, as dauer larvae are developmentally arrested, thin, and resistant to harsh conditions, features that are shared by infective-L3 larvae, although infective-L3 stage is an obligatory stage of the lifecycle while the *C. elegans* dauer stage is an alternative developmental pathway, which is environmentally induced by food limitation, unfavorable temperatures, and a high population density [1], [2]. A recent study showed that the dafachronic acid/DAF-12 receptor system is a conserved endocrine module, which controls entry into the dauer stage of free-living nematodes and the infective-L3 stage of parasitic nematodes such as *Strongyloides papillosus* [2].

After successful infection, parasitic nematodes not only have to adapt to the physiological environment of the host, such as high temperatures and low-oxygen pressure, but also have to endure the threat from the host immune response. Studies in the last few decades have shown that T-helper 2 (Th2) cells play a crucial role in protection against nematode parasites [3], [4]. Th2 cytokines activate bone marrow-derived cells, such as eosinophils, mast cells, and basophils, which then become involved in the expulsion of nematodes from the intestine, such as *Trichinella spiralis* and *Strongyloides venezuelensis*. On the other hand, protection against certain intraluminal nematodes such as *Nippostrongylus brasiliensis* has been suggested to be exerted not by bone marrow-derived cells, but through the effects of Th2 cytokines on non-bone marrow-derived cells, which may include changes in intestinal epithelial function, increased intestinal mucus secretion, and intestinal smooth muscle contractility [3]–[6]. However, little is known about the molecular responses that intestinal nematodes use to counteract host immunity.

In *C. elegans*, daf-2, insulin/insulin-like growth factor 1 receptor (IGF-1R) signaling system is one of the principal components affecting lifespan in *C. elegans*, and reduced IGF-1 signaling can lead to not only entry into larval diapause, but also a greater than two-fold increase in lifespan [7], [8]. The daf-16/forkhead transcription factor acts downstream of the IGF-1R signaling system and is negatively regulated by it. Interestingly, recent studies have disclosed that parasitic nematode infective-L3 larvae, such as those of *Strongyloides stercoralis*, ancylostomes, and *Haemonchus contortus*, express the daf-16/forkhead transcription factor [9]–[12], suggesting that parasitic nematodes exploit similar regulatory mechanisms to *C. elegans* to ensure their survival. The components that are important for enhanced longevity observed in *C. elegans* include the alpha-crystallin family of small heat shock proteins (Hsp), such as Hsp12.6, anti-reactive oxygen species (ROS) defense systems, and cellular phase II detoxication [13]. In the dauer stage of *C. elegans*, the expression levels of Hsp70 and Hsp90 are also upregulated, although not necessarily in a dauer-specific manner [14], [15]. In this respect, it is of interest to know whether parasitic nematodes also exploit Hsps to aid their survival in the two most critical stages of their lifecycle, the infective-L3 larva stage and adult worms under immunological pressure from the host.

Hsps are members of a highly conserved family of molecular chaperones that play multiple roles in vivo. There are a number of Hsps: large Hsps such as Hsp60, Hsp70, and Hsp90, as well as small Hsps with molecular masses of 12–43 kDa [16], [17]. Several Hsps have been characterized in parasitic nematodes: Hsp20 in *N. brasiliensis* [18]; Hsp20 and Hsp70 in *Haemonchus contortus* [19], [20]; Hsp70 in *Onchocerca volvulus* and *Parastrongyloides trichosuri* [21], [22]; and Hsp12.6, Hsp18, and Hsp90 in *Brugia* spp. [23]–[25]. Some of these nematode Hsps show stage-specific expression, suggesting that they play unique biological roles in certain stages of the lifecycle; however, because different lifecycles are exploited by different parasite species and rather few stages of their lifecycles have been investigated, the role of Hsps in the nematode lifecycle remains largely unknown. To overcome these problems, we investigated the detailed temporal gene expression patterns of 4 Hsps, Hsp12.6, Hsp20, Hsp70, and Hsp90, throughout the lifecycle of *N. brasiliensis*, including the worm expulsion stage. *Nb*-FKB-3 is a homolog of *C. elegans* FKB-3, which has been suggested to be involved in the synthesis of proline-rich cuticle collagens through its peptidyl-prolyl *cis-trans* isomerase activity [26]. Thus, the expression levels of *Nb*-FKB-3 were also determined as a molecular marker of body development.

*N. brasiliensis,* a rodent intestinal parasite, is a suitable model for studying clinically relevant hookworms because of their similar habitats and life cycles [27]. The development of *N. brasiliensis* proceeds through four larval stages (L1–L4) punctuated by molts to reproductively mature adult worms. Stages L1 to L3 are preparasitic. L3 is a critical stage during which development is arrested and then reactivated after percutaneous infection of the larvae into the host. After infection, the larvae migrate into the lungs and reach the intestine by 2–3 days postinfection (PI). In the intestine, L4 larvae quickly develop into adults. However, *N. brasiliensis* adults do not live long in immunocompetent hosts, as infections are normally terminated by 14 days PI by a T cell-dependent mechanism [3]–[6]. The present results showed that, among the Hsps examined, the expression of *Nb*-Hsp12.6 was upregulated in infective-L3 larvae and adults during the worm expulsion stage. Evidence suggested that *Nb*-Hsp12.6 is sensitively induced by host immunity, shedding new light on host-parasite interactions.

## Results

### Development of *Nippostrongylus brasiliensis*

**Figure 1:**
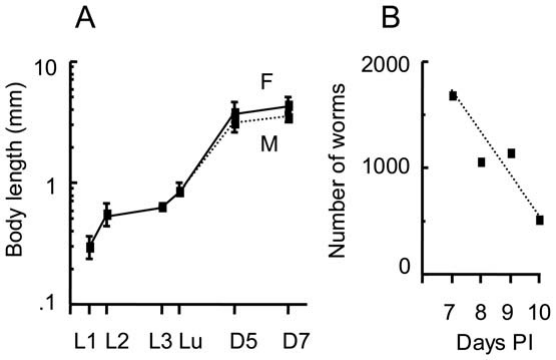
Development of *N. brasiliensis*. **A**. Development of *N. brasiliensis*. L1, 1st-stage larvae; L2, 2nd-stage larvae; L3, infective 3rd-stage larvae; Lu, lung stage-L3 larvae; D5, young adult worms collected at 5 days PI; D7, mature adult worms collected at 7 days PI. F, females; M, males. Data shown are mean and SD of 10–20 worms. **B**. Number of worms recovered from the intestine in the worm expulsion period.

To correlate the gene expression levels of Hsps with the development of *N. brasiliensis*, we briefly describe the morphological characteristics of worms in each stage of development, which were then subjected to temporal gene expression studies. Worms of the preparasitic stage (L1, L2, and infective-L3) were obtained from fecal culture, and worms of the parasitic stage (lung stage-L3, intestinal-stage L4, and adults) were obtained from experimentally infected SD rats. Morphological characteristics of each developmental stage are described in Table 1 and photographs are shown in Figures S1, S2, and S3 (the first to third supporting information figures). During the L1 and L2 stages, growth was rapid with body length almost doubling within 24 hours at 26°C, while the infective-L3 larvae were developmentally arrested (Figure 1A). After percutaneous infection in SD rats, the L3 larvae migrated to the lungs. The lung stage-L3 larvae recovered from the SD rats at 24 hours PI showed significant increases in body length (594 - 668 (mean: 638) µm *vs* 653–931 (mean: 780) µm for the infective-L3 and lung stage-L3 larvae, respectively), indicating that L3 larvae development was reactivated shortly after infection (Figure 1A). In the lungs, the third molt begins at about 32 hours, and most L4 larvae reach the intestine by the third day [28]. The L4 larvae recovered from the intestine at 3 days PI showed early sexual differentiation, which became more distinct together with the rapid growth of internal reproductive organs at 4 days PI. The 4th (final) molt begins from about 90–120 hours [29], [30]. The majority of worms recovered at 5 days PI showed almost fully developed genital organs. Intrauterine eggs were observed in some female worms obtained at 6 days PI and in virtually all female worms obtained at 7 days PI. The 7 day-old adults were slightly larger than the 5 day-old adults (Figure 1A), possibly due to the inclusion of some slowly developing L4 worms in the latter population. The worm burden in the small intestine of the SD rats started to decline from day 8 PI, and the majority of worms had been expelled by 10 days PI (Figure 1B).

**Table 1:** Morphological characteristics of the worms in each developmental stage of *N. brasiliensis*.

Stage	Morphological characters
L1	Cuticular annulation, indistinct; the esophagus, rhabditiform; the tail, gradually tapering and long (Figures S1 and S2).
L2	Cuticular annulation, distinct; the esophagus, rhabditiform; the tail, gradually tapering and long (Figures S1 and S2).
Infective L3	Cuticular annulation, distinct; the esophagus, tubular and slightly enlarged posteriorly; the tail, short and is provided with ventral protuberance (Figures S1 and S2).
Lung-stage L3	Intestinal cells, deeply pigmented with brown granules; other characters are similar to those in infective L3 (Figures S1 and S2).
L4	Cephalic cuticular expansion, visible; sexual differentiation, notable with formation of primitive vulva in females and primitive copulatory bursa in males; internal reproductive organs grow rapidly (Figure S3).
Adult	Reproductive organs, fully developed; cuticle, pinkish in shade (Figure S3)

### Hsps, FKB-3, and actin of *Nippostrongylus brasiliensis*

cDNA of the putative *Nb*-Hsp12.6, *Nb*-Hsp20, *Nb*-Hsp70, *Nb*-Hsp90, *Nb*-FKB-3, and *Nb*-actin were amplified by PCR using the primers listed in Table 2, and nucleotide sequences were determined. Although the target sequences did not include the entire coding region (except that of *Nb*-Hsp12.6), protein motif/domain searches revealed that the translated amino acid sequences of *Nb*-Hsp12.6, *Nb*-Hsp20, *Nb*-Hsp70, *Nb*-Hsp90, *Nb*-FKB-3, and *Nb*-actin showed unambiguous matches to the respective protein families (Table 3). Further, 3D models were constructed based on the amino acid sequences of *Nb*-Hsp12.6 and *Nb*-FKB-3 and these were compared with the 3D structure of *C. elegans* Hsp12.6 and FKB-3 (Figure S4: the 4th supporting information figure). A superpose analysis with the Molecular Operating Environment (MOE) program showed the 3D structural difference (RMSD) between *N. brasiliensis* and *C. elegans* to be 4.43 Å for Hsp12.6 and 7.34 Å for FKB-3 (Figure S5: the 5th supporting information figure), indicating that *Nb*-Hsp12.6 and *Nb*-FKB-3 were very similar to their *C. elegans* counterparts.

**Table 2:** Primers used in this study.

Target gene	F: Forward primer (5′- - 3′)R: Reverse primer (5′- - 3′)	Productsize (bp)^1^	References^2^	Sequences^3^
*Nb-*actin	F: CCGGATTTGCCGTCGATGA	435	EB390476	AB549202
	R: GAAGGATAGCATGAGGAAGG			
*Nb-*Hsp12.6	F1: ACCAAAGGAGATTGAGGTGAA	247^4^	EB185086	AB549204
	F2: GCCCTTGGGTTTAATTACCC	410^5^		AB608024
	R: ACGAAGCATTCATACAGCGTC			
*Nb-*Hsp20	F: AAGAGGTGATCAACGACGACR: CCGTGATTGTCTAACTCGGT	183	X71663	
*Nb-*Hsp70	F: AGGACAACAATCTGCTCGGAR: TGCTCGAACTCGTCCTTCTC	390	BM278875BM278839BM278966	AB549205
*Nb-*Hsp90	F: AAGGCGTTCATGGAAGCTCTR: TCTTCACGATCTCCTTGATGC	239	EB390876	AB549206
*Nb-*FKB-3	F: TTCACGTTCGTTCTTGGACGR: CCTTCCATACCGATTTCCAT	382	EB391004	AB549203

1. Amplified product size excluding primer regions.

2. BM278875, BM278839, and BM278966 (Harcus Y, Maizels RM. Sequence survey of *Nippostrongylus brasiliensis*. 2001), and EB390476, EB185086, EB390876, and EB391004 (Mitreva M, Fulton L, Becker M, Ronko I, Theising B, Martin J, Scott AL, McCarter JP, Wilson R. WashU Nematode EST Project. 2005).

3. Nucleotide sequences of the PCR products determined in the present study.

4. Product size using the F1-R primer set. For real-time PCR, this primer set was employed.

5. Product size using the F2-R primer set.

**Table 3:** Similarities of *N. brasiliensis* actin, Hsps, and FKB-3 to those of other nematodes.

Genes examined	Protein family^1^	Similar to^2^	Amino acid sequence		
			Identities^3^	Positives^4^	E-value
*Nb-*actin (AB549202)	Actin (PF00022)	Bm actin (EDP36330)	100	100	7e-82
*Nb-*Hsp12.6 (AB608024)	Acrystallin^5^ (PR00299)	Ce Hsp12.6 (Z68342)	59.8	74.8	8e-31
*Nb-*Hsp20 (X71663)	Heat shock protein 20 (IPR002068)	Hc Hsp20 (AY130968)	73.0	85.1	4e-56
*Nb-*Hsp70 (AB549205)	Heat shock protein 70 (IPR013126)	Dm Hsp70 (HM125969)	91.5	97.7	1e-64
*Nb-*Hsp90 (AB549206)	Heat shock protein 90 (IPR001404)	Hc Hsp90 (FJ717747)	97.5	98.7	8e-38
*Nb-*FKB-3 (AB549203)	Peptidyl-prolyl cis-trans isomerase, FKBP-type (IPR001179)	Ce FKB-3 (NM_072434)	71.1	79.7	2e-44

1 Protein family to which translated amino acid sequences of *N. brasiliensis* transcripts showed a ‘true’ match in the InterProScan sequence search.

2 The most similar sequence found by the BLAST search of the UniProt protein databases. Bm, *Brugia malayi*; Ce, *C. elegans*; Dm, *Dracunculus medinensis*; Hc, *Haemonchus contortus*.

3 Percent sequence identity.

4 Percent sequence similarity including changes at a specific position of an amino acid sequence that preserve the physico-chemical properties of the original residue.

5 Alpha crystallin/heat shock protein.

### Temporal gene expression patterns of *Nb*-Hsps throughout the lifecycle of *Nippostrongylus brasiliensis*

**Figure 2:**
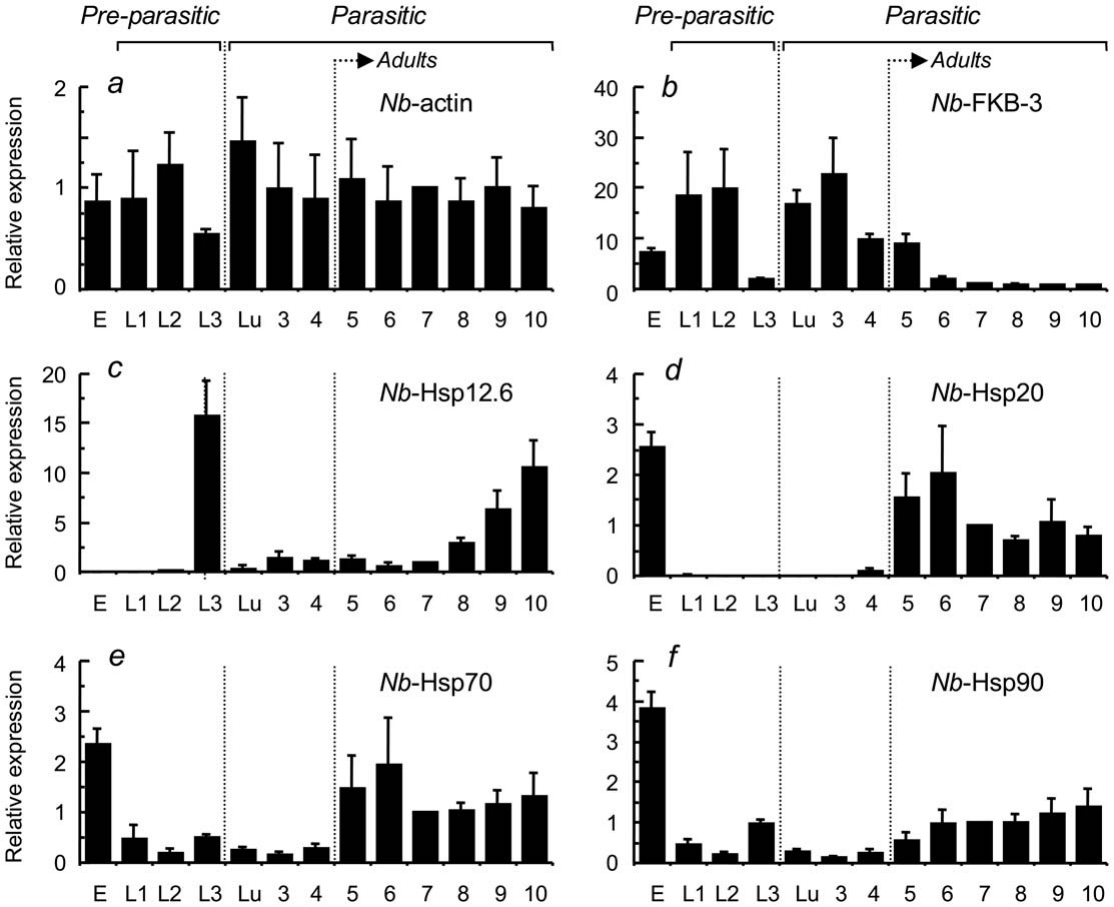
Temporal gene expression patterns during the lifecycle of *N. brasiliensis*. E, eggs; L1–L3, L1–L3 stage larvae in fecal culture; Lu, lung stage-L3 larvae; 3–12, intestinal worms recovered at the indicated days after infection. The pre-parasitic stage data are shown as the mean and SD of worm populations recovered from 3 batches of fecal cultures. The parasitic stage data are shown as the mean and SD of worm populations recovered from 3 SD rats. Except *Nb*-actin (*a*), the levels of which were normalized to RNA quantities, all gene expression levels (*b–f*) were normalized to those of *Nb*-actin. The values observed at 7 days PI are expressed as 1.0.

The transcription levels of *Nb*-Hsp12.6, *Nb*-Hsp20, *Nb*-Hsp70, *Nb*-Hsp90, *Nb*-FKB-3, and *Nb-*actin were determined throughout the lifecycle of *N. brasiliensis* by RT-real-time PCR. The worms subjected to this study were the same as those described in the above section, and the adult worms belonged to a mixed population of males and females. The expression levels of *Nb-*actin (normalized to the quantity of RNA) showed inter-stage variations of 0.54- to 1.56-fold when the level observed at 7 days PI was expressed as 1.0 (Figure 2a). To compare gene expression levels between the different stages of the lifecycle, all gene expression levels were normalized to those of *Nb-*actin.

*Nb*-FKB-3, a molecular marker of body development, was expressed at high levels in the L1, L2, lung-stage L3, and L4 larvae, but was markedly downregulated in the infective-L3 and mature adult worms, clearly showing that its expression was restricted to stages in which worms grow rapidly (Figure 2b).

*Nb*-Hsp12.6 showed a unique biphasic temporal expression pattern: its expression was upregulated in infective-L3 larvae as well as in adult worms older than 8 days PI (Figure 2c). Its expression in infective L3 larvae was quickly downregulated in the lung stage-L3 larvae, when the worms had resumed their development. In the intestinal stage, the expression of *Nb*-Hsp12.6 was marginal until day 7 PI. However, as worm expulsion proceeded from day 8 onward, *Nb*-Hsp12.6 expression was upregulated over time.

*Nb*-Hsp20 expression has been shown to be restricted to adult *N. brasiliensis* worms [18]. The present results confirmed those of the previous report and further showed that *Nb*-Hsp20 is expressed not only in adult worms, but also in the deposited eggs (Figure 2d). In contrast, *Nb*-Hsp70 and *Nb*-Hsp90 were expressed at readily detectable levels in all developmental stages. In the larval stages, the levels of *Nb*-Hsp70 and *Nb*-Hsp90 were higher in infective-L3 larvae than in other larval stages. The developmental stage that showed the highest expression levels of *Nb*-Hsp70 and *Nb*-Hsp90 were the adults and eggs. The expression levels of *Nb*-Hsp20, *Nb*-Hsp70, and *Nb*-Hsp90 did not change significantly during the worm expulsion period (Figure 2d-f).

### Gender differences of *Nb*-Hsps expression in adult worms

**Figure 3:**
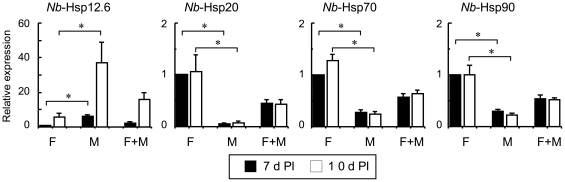
Gender differences in the Hsp gene expression levels of *N. brasiliensis* adult worms. F, females; M, males; F+M, a mixture of the same amounts of cDNA from males and females. The levels in the ‘F’ at 7 days PI are expressed as 1.0. Data shown are mean and SD of worms recovered from 4 SD rats. *P<0.05.

Gender differences in the gene expression levels of *Nb*-Hsps were examined in adult worms that were recovered at 7- and 10-days PI from SD rats. The expression of *Nb*-Hsp12.6 was approximately 6-fold higher in males than in females in both the 7 day- and 10 day-old worms. It should be noted that *Nb*-Hsp12.6 expression was upregulated after 10 days of infection in both males and females (Figure 3). In contrast, the expression levels of *Nb*-Hsp20, *Nb*-Hsp70, and *Nb*-Hsp90 were significantly higher in the female than the male worms. In particular, *Nb*-Hsp20 showed 17-fold and 13-fold higher expression levels in adult females than in males, at 7 and 10 days PI, respectively. As the eggs also expressed high levels of *Nb*-Hsp20, *Nb*-Hsp70, and *Nb*-Hsp90, the female-predominant expression of these Hsps may reflect, at least in part, the possession of large numbers of intrauterine eggs in female worms.

### Expression of *Nb*-Hsps in adult worms in athymic rats and mast cell-deficient *Ws/Ws* rats

**Figure 4:**
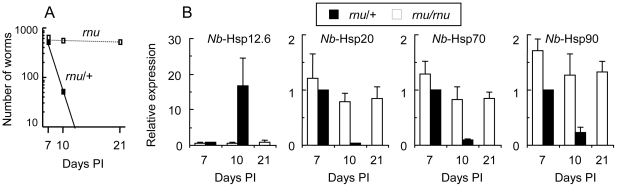
Hsp gene expression in adult worms in athymic rats. **A**. Worm burdens in the small intestine of athymic nude (*rnu/rnu*) (n = 3/group) and euthymic *rnu*/+ rats (n = 3/group) after infection with 1,000 infective-L3 larvae. **B**, Hsp-gene expression levels of worms recovered from athymic and euthymic rats. For day 21, as all worms had been expelled from *rnu*/+ rats, only data from *rnu/rnu* rats are shown. Gene expression levels were normalized to those of *Nb*-actin. The levels in worms recovered from euthymic rats at 7 days PI are expressed as 1.0.

*Nb-*Hsp12.6 expression showed progressive upregulation during worm expulsion from the intestine of immunocompetent hosts. To determine whether the upregulation of *Nb-*Hsp12.6 expression in adult worms was associated with aging or was induced by host immunological reactions, the expression levels of *Nb*-Hsp12.6 were examined in adults that had infected athymic *rnu/rnu* rats and their littermate euthymic *rnu*/+ rats with an F-344 background. Worm rejection did not occur in the athymic rats until at least 21 days PI, while in the euthymic rats, approximately 90% of the worms had been expelled from the intestine by 10 days PI, and no worms were recovered at 21 days PI (Figure 4A). Adult worms recovered from the athymic rats at 10 and 21 days PI showed only marginal *Nb*-Hsp12.6 expression, while those collected from euthymic rats at 10 days PI exhibited markedly upregulated *Nb*-Hsp12.6 expression (Figure 4B). On the other hand, 10 day-old adult worms in euthymic rats showed marked reductions in the levels of *Nb*-Hsp20, *Nb*-Hsp70, and *Nb*-Hsp90 compared to those in the athymic rats. The finding that the euthymic *rnu*/+ F-344 rats induced markedly suppressed *Nb*-Hsp20, *Nb*-Hsp70, and *Nb*-Hsp90 expression in worms in the terminal parasitic stage differed from those observed in the SD rats, in which the expression levels of the above genes did not change significantly (Figure 2). We also determined *Nb*-Hsps levels normalized to *Nb*-globin b expression. The results were comparable to those normalized to *Nb*-actin, despite small differences (Figure S6, the 6th supporting information figure).

**Figure 5:**
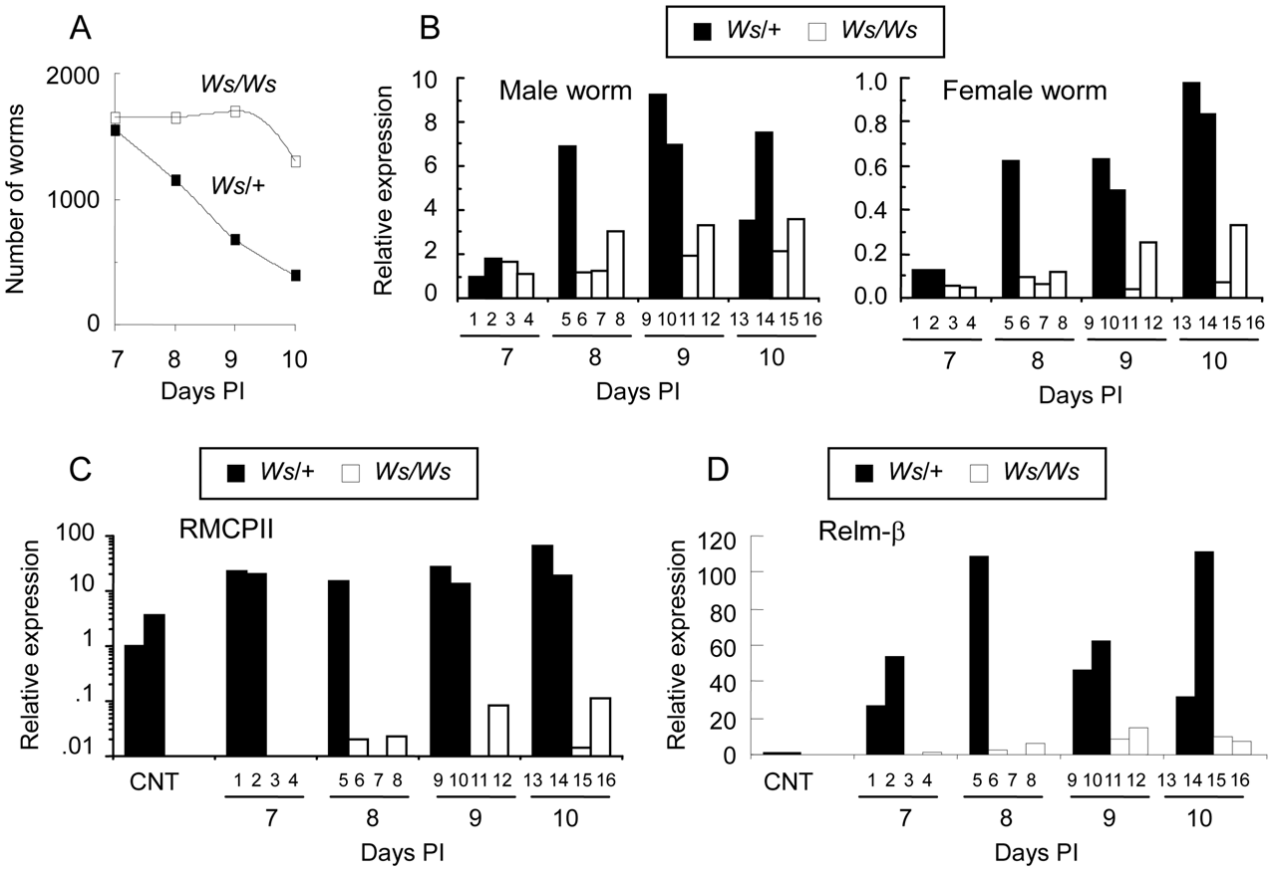
Hsp gene expression in adult worms in mast cell-deficient rats. **A**. Mean worm burdens in the small intestine of mast cell-deficient *Ws/Ws* and the control *Ws*/+ rats after infection with 2,000 infective-L3 larvae. **B**. *Nb*-Hsp12.6 gene expression levels in male and female worms recovered from *Ws/Ws* and *Ws*/+ rats. Individual data are shown with rat ID numbers (1–16) beneath the columns. The expression level in the male worms from rat No. 1 is shown as 1.0. **C**. Gene expression levels of RMCP II in the intestinal mucosa of *Ws/Ws* and *Ws*/+ rats. The expression level in one of the uninfected *Ws*/+ rats (CNT) is shown as 1.0. **D**. Gene expression levels of Relm-β in the intestinal mucosa of *Ws/Ws* and *Ws*/+ rats. The level in one of the uninfected *Ws*/+ rats (CNT) is shown as 1.0.

To determine the relationship between mast cell activation and *Nb*-Hsp12.6 expression, the expression levels of *Nb*-Hsps were examined in worms that had infected mast cell-deficient *Ws/Ws* rats and heterozygous *Ws*/+ rats, which have normal numbers of mast cells. Due to the low yield (poor reproductivity) of these rats, the experiment was conducted with a limited number of animals, and data from individual animals are presented in Figure 5. Worm expulsion was delayed in *Ws/Ws* rats compared with that in *Ws*/+ rats, in which the worms were expelled progressively from day 7 to day 10 (Figure 5A). *Nb*-Hsp12.6 expression was upregulated in both male and female worms during the worm expulsion period in *Ws*/+ rats. In the *Ws/Ws* rats, a small increase in the level of *Nb*-Hsp12.6 was also observed, especially at 9 and 10 days after infection; however, the overall increase in *Nb*-Hsp12.6 expression in male and female worms in *Ws/Ws* rats was lower than that in *Ws*/+ rats (Figure 5B). It has been reported that a small but significant number of mast cells develop in the small intestine of *Ws/Ws* rats after *N. brasiliensis* infection [31]. To determine the level of mast cell activation, the expression levels of mucosal mast cell-specific rat mast cell protease (RMCP) II in the intestinal mucosa of animals from which worms had been recovered were examined. As shown in Figure 5C, markedly upregulated RMCP II expression was observed in *Ws*/+ rats. In *Ws/Ws* rats, low level-RMCP II expression was found in some of the animals after infection. It should be noted that *Ws/Ws* rats that displayed upregulated RMCP II expression (for instance, rats 12 and 16 in Figure 5B) harbored worms that also showed upregulated *Nb*-Hsp12.6 expression.

Relm-β is produced in and secreted from goblet cells [32], [33]. The expression levels of Relm-β in the intestinal mucosa of *Ws/*+ rats showed marked upregulation as early as day 7 PI compared to those in the uninfected controls, whereas, the levels of Relm-β in the nematode-infected *Ws/Ws* rats were markedly lower than those in the *Ws/*+ rats (Figure 5D). *Nb*-Hsp20, *Nb*-Hsp70, and *Nb*-Hsp90 expression were also examined in adult worms recovered from *Ws/Ws* and *Ws*/+ rats. There were no significant changes in the levels of these Hsps between day 7 and day 10 PI in worms recovered from *Ws/Ws* or *Ws*/+ rats (data not shown).

### Expression of *Nb*-Hsps in adult worms transplanted into a new host

**Figure 6:**
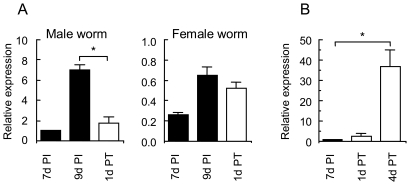
Expression of *Nb*-Hsp12.6 in adult worms transplanted into the intestine of a new host. **A**. Transplantation of 9 day-old adult worms into the intestine of naive rats. Nine-day old (9 d PI) male and female worms (200 each) recovered from donor SD rats were transplanted into the intestine of naive SD rats (n = 3/group). The worms were recovered at 24 hours post-transplantation (1 d PT). **B**. Transplantation of 7 day-old adult worms into the intestine of naive rats. Seven-day old adult worms (7 d PI) (a mixed population of males and females) recovered from the donor rats were transplanted into the intestine of naive SD rats (n = 3/group). The worms were recovered at 1 and 4 days after transplantation (1 d PT and 5 d PT, respectively). *Nb*-Hsp12.6 expression levels in the 7 d PI male worms (**A**) and the mixed population of 7 d PI male and female worms (**B**) are expressed as 1.0. Data shown are the mean and SE of 3 rats.

If *Nb*-Hsp12.6 is inducible by host immunity or pathophysiological changes, its expression may be reversible. To confirm this, male and female worms (200 each) that had been recovered from donor SD rats at 9 days PI were separately transplanted into the intestine of naive SD rats via a gastric tube. Thirty - 45% of the administered male worms and 45 - 60% of the administered female worms had established themselves in the intestine of the recipient rats at 24 hours after transplantation. The expression of *Nb*-Hsp12.6 in the male worms was significantly downregulated at 24 hours after transplantation compared to the pre-transplantation levels, while that of *Nb*-Hsp12.6 in female worms was not (Figure 6A). The expression levels of *Nb*-Hsp20, *Nb*-Hsp70, and *Nb*-Hsp90 were not significantly altered after transplantation (data not shown).

If specific immunity result in the upregulation of *Nb*-Hsp12.6 expression, it is expected to take some time, possibly >7 days after infection, for *Nb*-Hsp12.6 to be upregulated. To clarify this, a mixed population of approximately 400 male and 400 female worms recovered from the donor SD rats at 7 days PI were transplanted into the intestine of naive SD rats via a gastric tube. Approximately 20–50% and 25–62% of the administered worms had established infection in the small intestine of the recipient rats at 1 and 4 days after transplantation, respectively, with approximately the same sex ratio. Although no adult worms showed significantly upregulated *Nb*-Hsp12.6 expression at 1 day post-transplantation, the worms recovered at 4 days after transplantation showed markedly upregulated *Nb*-Hsp12.6 expression (Figure 6B). These results suggested that fully-developed acquired immunity is not necessarily required for the activation of *Nb*-Hsp12.6.

### Expression of *Nb*-Hsps in infective-L3 larvae

**Figure 7:**
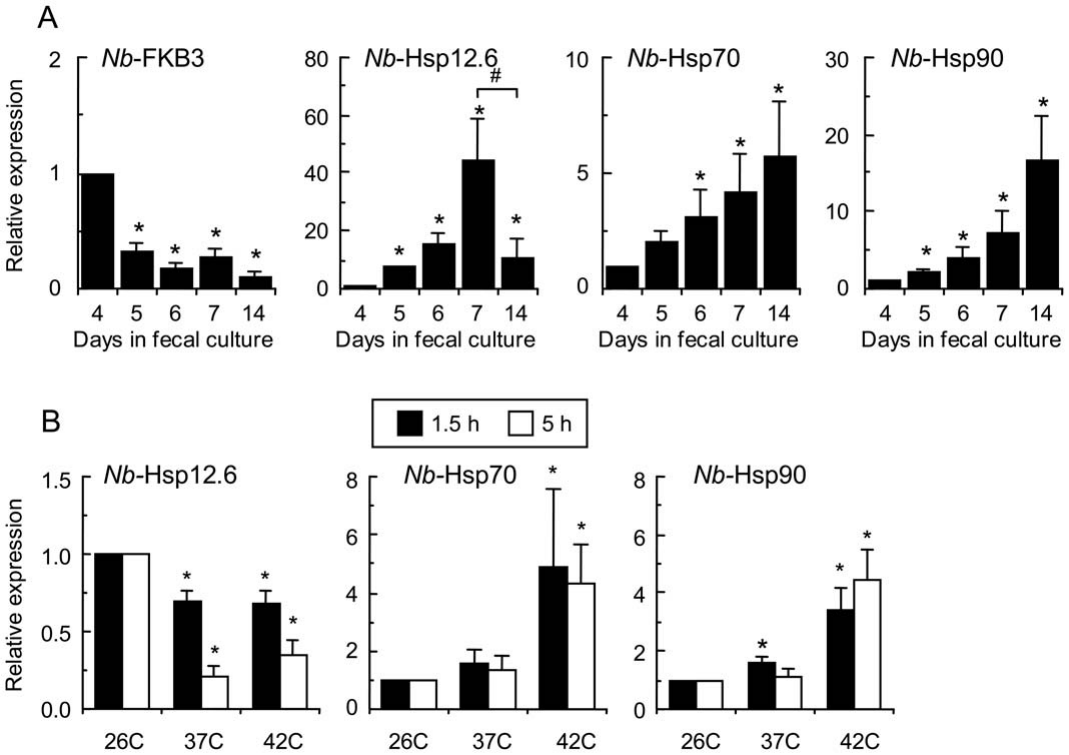
Hsp gene expression in the pre-parasitic stages of *N. brasiliensis*. **A**. Temporal gene expression patterns of larvae during 4 to 7 days fecal culture at 26°C. Levels at 4 days fecal culture are expressed as 1.0. Data shown are the mean and SD of larval populations from 4 fecal cultures derived from 4 host animal feces. *P<0.05 compared with the levels in 4 day-old fecal culture. **B**. Heat shock responses of infective L3 larvae. Infective-L3 larvae in 7 day-old fecal culture (26°C) were incubated at 37 or 42°C for 1.5 or 6 hours. The expression levels in the larvae incubated at 26°C are expressed as 1.0. Data shown are the mean and SD of larvae from 4 fecal cultures derived from 4 host animal feces. *Significantly different from the levels of larvae incubated at 26°C (*P*<0.05).

Infective-L3 larvae showed not only the potent upregulation of *Nb*-Hsp12.6 expression, but also comparatively high expression levels of *Nb*-Hsp70 and *Nb*-Hsp90. To further confirm these results, we examined the expression of *Nb*-Hsps in the larvae recovered at 4–7 days after the start of fecal culture, as well as those recovered after 14 days of fecal culture. The development of eggs into infective-L3 larvae generally takes 4 to 5 days [30]. As shown in Figure 7A, *Nb*-FKB-3 showed a high expression level in 4 day-old larvae in fecal culture, but its expression was significantly downregulated in larvae older than_5 days, suggesting that the 5 day-old larvae had almost completed their development.

The expression levels of *Nb*-Hsp12.6, *Nb*-Hsp70, and *Nb*-Hdp90 were significantly upregulated in the 5 day-old larvae compared to those in the 4 day-old larvae, and the levels increased progressively until day 7 (Figure 7A). These results suggested that the expression levels of *Nb*-Hsp12.6, *Nb*-Hsp70, and *Nb*-Hdp90 are developmentally regulated, at least in part, because the fecal culture conditions generally remain stable until at least day 7. However, the feces gradually desiccate after 7 days. In the 14 day-old larvae, the expression levels of *Nb*-Hsp12.6 were significantly lower than those in the 7 day-old larvae, whereas the levels of *Nb*-Hsp70 and *Nb*-Hsp90 were higher than those in the 7 day-old larvae, suggesting a possible effect of fecal-culture deterioration on the expression of these *Nb*-Hsps.

We further examined the heat responsiveness of *Nb*-Hsps in infective-L3 larvae. When 7 day-old infective-L3 larvae, which had been maintained at 26°C, were exposed to 37°C or 42°C, the expression levels of *Nb*-Hsp12.6 decreased significantly, whereas the expression levels of *Nb*-Hsp70 and *Nb*-Hsp90 showed significant upregulation at 42°C, although at 37°C, the increases in the levels of *Nb*-Hsp70 and *Nb*-Hsp90 were only marginal (Figure 7B).

## Discussion

The present studies clarified the detailed temporal expression patterns of *Nb*-Hsps throughout the lifecycle of *N. brasiliensis* and found that they are closely correlated with development, niche transition, and worm expulsion. The most interesting *Nb*-Hsp temporal expression patter was that of *Nb*-Hsp12.6: it was upregulated in the infective-L3 larvae and the adult worms during the worm expulsion period. The expression of *Nb*-Hsp12.6 during the last stage of parasitism appeared to be specific to the worms that were ready to be expelled from the small intestine, inasmuch as the upregulation occurred in worms that had infected immunocompetent hosts, but not in worms that had infected in permissive-athymic rats. The worms recovered from the small intestine during the worm expulsion period did not enter into an irreversible dying process as they were able to reestablish themselves when transplanted into uninfected rats. However, previous studies showed that when expulsion phase and normal worms (e.g. worms recovered at 7 days PI) were transplanted into a new host, the former worms reestablished themselves less efficiently and produced fewer eggs than the latter worms, suggesting that the worms in the expulsion phase are somehow ‘damaged’ by immunity [27]. It is also known that male worms are more tolerant to immune expulsion than female worms [27]. In this respect, it is interesting to note that the male worms expressed approximately 6-fold higher *Nb*-Hsp12.6 levels than the females. The expression of *Nb*-Hsp12.6 in the ‘damaged’ male worms was reversible in the new host, while that of the ‘damaged’ females was not. The reason for this gender difference is not clear, but it appears that the damaged female worms did not have the same abilities to recover in the new host as the male worms.

The intestinal mucosa exhibits a number of immunopathological changes following *N. brasiliensis* infection, including mucosal mastocytosis, goblet cell hyperplasia, mucus hypersecretion, secretion of non-mucus peptides such as Relm-β, alterations in the sugar chains of intestinal epithelial membrane glycoproteins, and increased smooth muscle contractility [3], [5], [34]. The key effector molecule(s) that induce the termination of *N. brasiliensis* parasitism are disputed, while recent reports indicated that Relm-β, which is produced in and secreted from goblet cells, is most likely to be the effector molecule responsible for protection against lumen dwelling nematodes in mouse models [32], [33]. The role of mast cells in protection against intramucosal and/or intraepithelial nematodes such as *Trichinella spiralis* and *Strongyloides* spp. has been well documented [5]. However, mast cells are not essential for the expulsion of *N. brasiliensis* at least in mouse models, despite that significant intestinal mastocytosis occurs following *N. brasiliensis* infection [3], [5]. The present results showed a delay in the start of worm expulsion in *Ws/Ws* rats compared to that in control *Ws*/+ rats, while too few animals were used to determine the significance of differences. Nevertheless, the results showed striking correlations between the levels of mucosal RMCP II and the levels of *Nb*-Hsp12.6, as well as between those of Relm-β and *Nb*-Hsp12.6. Athymic rats do not develop intestinal mastocytosis upon infection with *N. brasiliensis*, and the response of Relm-β in athymic rats is significantly lower than that in euthymic rats [35]. These results suggest that pathophysiological changes in the intestinal niche, including mast-cell activation and Relm-β secretion, initiated the upregulation of *Nb*-Hsp12.6 expression. It was surprising that the *Ws/Ws* rats showed a low (or slow) response of Relm-β expression to nematode infection compared to that of *Ws*/+ rats because the only reported mutation in *Ws/Ws* rats was a 12-base deletion in the tyrosine kinase domain of the c-*kit* receptor, which is indispensable for the induction of mast cells by stem cell factor [36]. Recently, Lin^-^ *c-kit* ^+^ innate immune cell populations, such as multi-potent progenitor type 2 (MPP^type2^) cells and nuocytes, have been identified. These cells are generated in response to helminth infections and function as initiators of Th2 cytokine responses, including the production of mucin by goblet cells [37]–[39]. In this respect, it remains to be elucidated whether *Ws/Ws* rats with a defective *c-kit* receptor have a deficiency in *c-kit* ^+^ MPP^type2^ cells or *c-kit* ^+^ nuocytes.

The transplantation of 7 day-old adult worms into the intestine of naive rats induced the significant upregulation of *Nb*-Hsp12.6 expression as early as 4 days after transplantation, suggesting that the activation of *Nb*-Hsp12.6 does not necessarily require acquired immunity. After *N. brasiliensis* infection, RMCP II and Relm-β expression were upregulated as early as 7 days PI in normal rats; i.e., before acquired immunity had fully developed. Relm-β expression is reported to be greatly increased in the small intestine of *N. brasiliensis*-inoculated mice soon after the larvae had migrated to that organ [33]. It has also been indicated that there are 2 types of mucosal mast cell activation, prompt IL-18-dependent (innate type-2) activation and late Th2 cell-dependent (acquired type-2) activation [40]. Overall, it seems likely that some form of innate immunity is also able to initiate the activation of *Nb*-Hsp12.6 expression.

Contrary to the immunity-responsive activation of *Nb*-Hsp12.6, its expression in infective L3-larvae appeared to be regulated developmentally, inasmuch as its expression levels started to increase in coordination with the downregulation of *Nb*-FKB-3 expression, while the expression of *Nb*-Hsp12.6 was downregulated within 24 hours of infection in rats together with the simultaneous upregulation of *Nb*-FKB-3 expression. In *C. elegans*, the expression of small Hsps is induced by reduced IGF-1R signaling, whereas the expression of FKB-3 is positively regulated by signaling through the IGF-1R pathway [41], [42], suggesting that similar regulatory mechanisms control the entry of larvae into the infective-L3 stage. However, we have no direct evidence that the IGF-1R pathway controls the activation of *Nb*-Hsp12.6 expression in infective-L3 larvae and/or adult worms in the last stage of parasitism, and other pathways could well be critical.

Small Hsps, which range in size from 12 to 43 kDa, constitute a diverse family and are less conserved between organisms than other heat shock protein genes, but they share a common domain, α-crystalline, which is found in the vertebrate lens protein [43], [44]. Small Hsps have been reported to exist in several parasitic nematodes: *Brugia malayi*-Hsp18 in L4 larvae and adult worms but not in microfilariae; and *H. contortus*-Hsp20 in L3, L4, and adult worms [20], [23]. A common feature of small Hsps is their formation of large oligomeric complexes and their ability to prevent the aggregation of proteins [17], [45]. Some small Hsps appear to bind to actin and intermediate filaments [46]. Interestingly, Hsp27 and alpha-crystallin have been reported to enhance the survival of cells by conferring increased stability to actin fibers [47], [48]. In *C. elegans,* Hsp12.6 and 12.3 were expressed in dauers but not in non-dauer L3 control, and in long-lived daf-2 mutant adults in higher levels than in control adults, and the overexpression of small Hsps conferred lifespan extension in *C. elegans*, although small Hsps are only one of several components of the longevity system of *C. elegans* [13]–[15], [49]. These features of small Hsps suggest that *Nb*-Hsp12.6 also functions as a molecular chaperone that helps *N. brasiliensis* to survive deleterious environmental conditions both outside and inside of the host.

*Nb*-Hsp20 was expressed in both adult worms and eggs. It is possible that immunity against adult worms triggered its expression. However, its expression levels did not change when 9 day-old adults or 7 day-old adults were transplanted into a new host. Thus, the expression of *Nb*-Hsp20 appears to be regulated according to a strict developmental program, independently of a variety of stress stimuli, as suggested previously [18]. *Nb*-Hsp70 and *Nb*-Hsp90 were expressed throughout the nematode's lifecycle, suggesting that they play indispensable roles in cell maintenance at all stages of development. In infective-L3 larvae, not only *Nb*-Hsp12.6, but also *Nb*-Hsp70 and *Nb*-Hsp90 were upregulated, which was similar to findings in *C. elegans* [14], [15]. *Nb*-Hsp70 and *Nb*-Hsp90 showed further upregulation in aged infective-L3 larvae and under heat stresses, suggesting their involvement in the survival of infective-L3 larvae in harsh environments. Contrary to the finding for *Nb*-Hsp12.6, host immunity did not markedly affect the expression of *Nb*-Hsp70 or *Nb*-Hsp90 in worms that had infected SD rats, whereas marked reductions in their expression levels were found in 10 day-old worms in euthymic *rnu*/+ F-344 rats. Although the reason for this discrepancy is unclear, one possible explanation is that the immune reactions of *rnu*/+ F-344 rats affected the viability of the worms more severely than those of the SD rats. This possibility is in accordance with earlier observations that F-344 rats rejected all worms from the intestine after a certain period of time, while in other rat strains such as SD and BN, a small proportion of worms escaped rejection and persisted for a long period of time [50].

In conclusion, we showed that different Hsps are uniquely expressed at different stages of the lifecycle of *N. brasiliensis* and might play diverse roles in nematode survival under various kinds of stress. In particular, *Nb*-Hsp12.6 responded to immune stress, highlighting a new aspect of host-parasite interactions.

## Materials and Methods

### Ethics Statement

This study was carried out in strict accordance with the recommendations in Guidelines for Proper Conduct of Animal Experiments of the Science Council of Japan. The protocol was approval by the Animal Experiment Committee of Kyoto Prefectural University of Medicine (Permit Number: M22-7). Euthanasia was performed by an overdose of pentobarbital, and all efforts were made to minimize suffering.

### Nematode and infection

The strain of *N. brasiliensis* used in the present study has been maintained in our laboratory by passage in Sprague-Dawley (SD) rats via the subcutaneous inoculation of 2000 L3 larvae every 2 weeks. Ten - 12 week-old male SD rats were purchased from Shimizu Laboratory Supplies Co., Ltd., Kyoto, Japan, and 8-week-old female *rnu/rnu* (F344/N Jcl-rnu/rnu) rats and their littermate *rnu*/+ rats (F344/NJcl-*rnu*/+) were obtained from Clea Japan Inc., Tokyo. Male and female *Ws/Ws* rats and their littermate *Ws*/+ rats were produced in our laboratory as described previously [51]. For experimental infection, 2,000 L3 larvae were subcutaneously injected into SD, *Ws/Ws,* and *W*s/+ rats, and 1,000 L3 larvae were injected into *rnu/rnu* and *rnu*/+ rats.

### Collection of preparasitic-stage larvae

Feces collected from SD rats at 7 days PI were subjected to fecal culture using the filter paper test tube method at 26°C. L1-predominant and L2-predominant larval populations were recovered from 32 hour- and 56 hour-fecal cultures, respectively, and infective-L3 larvae were obtained from 7 day-old fecal cultures. In some experiments, larvae were recovered at different time intervals. For the larvae collection, test tubes containing filter paper covered with a fecal smear were filled with distilled water at 32C to allow the larvae in the feces to enter the water. After 60 seconds, the larvae in the water were collected and washed 3 times with distilled water. Then, 10,000–20,000 larvae/batch were subjected to RNA extraction. The L1 stage-predominant larval population consisted of worms measuring 207–396 µm in length and 10–21 µm in width (mean: 302×15 µm, n = 20), possibly reflecting uncoordinated egg-hatching time in fecal cultures. A few larvae with a body length of >350 µm were ensheathed, indicating an approaching molting. The L2 stage-predominant larval population consisted of worms measuring 347–673 µm by 19–32 µm (mean: 560×27 µm, n = 20). Within this larval population, some small larvae with body lengths of about <400 µm and large larvae of >600 µm were ensheathed, while the majority of larvae between 400 and 600 µm were not ensheathed. These and other morphological characteristics described in Table 1 indicated that the majority of this population was composed of stage L2 larvae. The infective-L3 larvae measured 594–668 by 25–31 µm (mean: 638×28 µm, n = 20).

### Collection of parasitic-stage larvae and adults

To minimize stresses during sampling, all procedures were carried out as quickly as possible, including the saline incubation method, which was minimized to 20 minutes. The worms were immersed in cold Trizol reagent (Life Technologies, Rockville, MD) within 30 min of the start of sampling, except when the male and female worms were separated under a stereoscopic microscope, which took an additional 30 minutes. Lung-stage L3 larvae were recovered from the lungs of SD rats at 24 hours after infection. The lungs removed from the animals were cut into approximately 1 mm cubic blocks and subjected to incubation in saline at 37°C. The recovered larvae measured 653–931 µm by 25–37 µm (mean: 780×31 µm, n = 10), and none of them were ensheathed. Twohy [28] described that the third molt begins in the lungs at about 32 hours and that there is no further growth until the larvae migrate into the intestine, with mean lengths of 0.943 mm and 0.957 mm at 32 and 41 hours after infection, respectively. From the size of the larvae and other characteristics described in Table 1, we concluded that the majority of larvae recovered from the lungs after 24 hours belonged to the L3 stage. Intestinal-stage worms were collected from SD rats daily from 3 to 10 days PI. In brief, a small intestinal segment at 0–40 cm from the pyloric ring, where the majority of worms parasitize, was removed, cut longitudinally, and subjected to saline incubation at 37°C. From 9 to 12 days PI, when worms are gradually expelled from the intestine, the intestinal segment at 0–60 cm from the pyloric ring was used for worm collection. Except in some experiments, the recovered adult worms were not separated into males and females. In the SD rats, approximately 400–800, 1,000–1,500, and 50–800 worms were collected from day 3 to day 4, day 5 to day 8, and day 9 to day 10 PI, respectively.

### Collection of eggs

Eggs were collected via the cultivation of adult female worms *in vitro*. In brief, approximately 800 female worms recovered 7 days after infection were incubated in 10 ml of phosphate buffered saline with 100 U/ml penicillin and 100 µg/ml streptomycin in a 25 square cm tissue-culture flask for 24 hours at 37°C, during which large numbers of eggs were laid. The medium containing the deposited eggs was sieved through a # 120 mesh, and eggs were collected by centrifugation. The majority of the eggs collected belonged to the morula stage.

### Adult worm transplantation

In the first experiment, 9 day-old worms were recovered from the intestine of 3 donor rats, and 200 male and 200 female worms were separately transferred into the intestine of 3 naive SD rats using a gastric tube. The transplanted worms were recovered from the intestine of the recipient rats 24 hours later. Approximately 60, 80, and 90 male worms were recovered from 3 rats, and 90, 100, and 120 female worms were recovered from 3 rats. In the second experiment, adult worms, which were recovered from 6 donor rats at 7 days PI, were transplanted into the intestine of 6 naive SD rats (a mixed population of 400 males and 400 females/rat) using a gastric tube. The recipient animals were autopsied at 1 and 4 days after transplantation. Approximately 160, 300, and 400 worms were recovered from 3 rats at 24 hours after transplantation, and 200, 400, and 500 worms were recovered from 3 rats at 4 days after transplantation. The sex ratio of the worms recovered from the recipient rats was approximately equal.

### Heat shock treatment of *N. brasiliensis* L3-stage larvae

Seven day-old fecal culture test tubes, which were kept at 26°C and contained infective-L3 larvae, were transferred to and maintained in incubators at 37°C or 42°C. After 1.5 or 5 hours, infective-L3 larvae were recovered.

### RNA extraction and cDNA synthesis

Total RNA was extracted with TRIZOL reagent (Life Technologies, Rockville, MD) according to the standard protocol provided by the manufacturer. cDNA synthesis was carried out using 0.1-µg aliquots of RNA from the eggs and L1-stage larvae, 0.2-µg aliquots of RNA from the lung-stage larvae, and 2-µg aliquots of RNA from all other worm stages in 20 µl of reverse transcription buffer containing 5 mM MgCl_2_, 1 mM of each deoxynucleoside triphosphate, 1 U of RNase inhibitor per µl, 0.25 U of avian myeloblastosis virus reverse transcriptase per µl, and 0.125 µM oligo(dT) primer (Takara RNA LA PCR kit; Takara Biomedicals, Osaka, Japan) at 42°C for 50 min.

### PCR amplification and sequencing

PCR was carried out using the primers described in Table 2. These primers were designed based on nucleotide sequences in public DNA databases. Both strands of the PCR amplicons were directly sequenced, and the nucleotide sequences were deposited in DNA databases under the accession numbers indicated in Table 2. The translated amino acid sequences were subjected to protein pattern and motif searches using InterProScan, FingerPrintScan, and Prosite.

### Homology Modeling of *Nb*-HSP-12.6 and *Nb*-FKB-3

The X-ray structure of template proteins was obtained from the RCSB Protein Data Bank: PDB ID 2 bol and 1q6h, for a small heat shock protein and a cis/trans peptidyl-prolyl isomerase, respectively. Sequence alignments were conducted using the ClustalW program. Following the alignments, 3D-models of *Nb*-HSP12.6 and *Nb*-FKB-3 were generated with MODELLER 9v8 software [52] using default parameters. The predicted 3D structures were deposited in the Protein Model Database (PMDB) with accession numbers PM0077219 and PM0077220. The model structures were soaked into water molecules and subjected to a molecular mechanics (MM) calculation with the AMBER99 force field until the root mean square (RMS) gradient was 0.01 kcal/mol/Å. Then, 100 ps molecular dynamics (MD) simulations at 300 K were performed. MM and MD simulations were performed using the Molecular Operating Environment (MOE) program, Version 2010.10 (Chemical Computing Group Inc., Montreal, Quebec, Canada). The resulting structures of *Nb*-Hsp12.6 and *Nb*-FKB-3 were compared with *C. elegans* HSP-12.6 (ModBase model id: 85be06ed48338ef7f27bad71c2223a16) or *C. elegans* FKB-3 (ModBase model id: ac47f3238b9ebb90bbb0c6fc85eb7a02) by the superpose function of the MOE.

### Real-time PCR

One-microliter aliquots of the synthesized cDNA were mixed with Sybr Green PCR master mix (Applied Biosystems, Foster City, CA) and appropriate primers and were subjected to amplification using a real-time PCR system 7300 (Applied Biosystems). The primers used are shown in Table 2. The specificity of each amplified product was confirmed by dissociation analysis, which produced a single sharp dissociation peak, the absence of the amplified product without reverse transcription, and the appearance of a band of the expected size on electrophoresis of the amplified product. For quantification, a standard curve of the amplification threshold cycle (Ct) for each gene against log ng total RNA was created by serially diluting the cDNA sample with the highest Ct value in undiluted conditions. The Ct value for each sample was then converted to an RNA quantity by referring to the corresponding standard curve. All quantified values were normalized to those of *Nb*-actin (quantified value for the target gene/quantified value for *N*b-actin). As an alternative house keeping gene, levels of *Nb*-globin b, which is expressed only during the parasitic stages of the lifecycle of *N. brasiliensis* [53], were also determined by real-time PCR using 5′-CTTCTGCTCTCAGTCCACAT-3′ and 5′-TGCTGGCATTCGTCGTTGAA-3′.

### Real-time PCR for RMCP II and Relm-β

Mucosal scrape specimens were obtained from the small intestine of *Ws/Ws* and *Ws*/+ rats with or without infection with 2,000 infective-L3 larvae. Total RNA was extracted with TRIZOL reagent, and cDNA was synthesized as described above. The expression levels of RMCP II and Relm-β were determined by real-time PCR using the primers 5′-TCCTACCTCGTATACACTGA-3′ and 5′- TTGCATCTGGATGCCCATAA-3′ for RMCP II, and 5′- TTCCTTCTCTCGCTGATGGT-3′ and 5′- GCAGTGGCAAGTAGTTCCAT-3′ for Relm-β. The quantified values as described above were normalized to those of Gapdh, which was also determined by real-time PCR using the primers 5′-CATCATCCCTGCATCCACTG-3′ and 5′-CAAAGGTGGAGGAATGGGAG-3′.

### Statistical analyses

The Student's t test was employed to determine statistical significance. *P* values of <0.05 were considered significant.

## Supporting Information

Figure S1 Anterior body of L1 (A), L2, (B), infective L3 (C), and lung-stage L3 (D) larvae of *N. brasiliensis*. Arrows indicate the esophageal-intestinal junction.
(TIF)

Figure S2 Posterior body of L1 (A), L2 (B), infective L3 (C), and lung-stage L3 (D) larvae of *N. brasiliensis.* Arrowheads, anus; arrows, small protuberance.
(TIF)

Figure S3 L4 larvae (A–C) and adult worms (D, E) of *N. brasiliensis*. L4 larvae were those at 3 days PI and adult worms were those at 6 days PI. A, anterior body, showing the cephalic cuticular expansion (ce). B, posterior end of a female worm, showing the primitive vulva (pv) and anus (a). C, posterior end of a male worm, showing the primitive copulatory bursa (pcb). D. middle of a female body, showing intrauterine eggs. E, posterior end of a male worm, showing copulatory spicules (s) and copulatory bursa (cb).
(TIF)

Figure S4 3D structure of Hsp12.6 and FKB-3.
(TIF)

Figure S5 Structural superpositions of *N. brasiliensis* HSP12.6 and *C. elegans* HSP12.6 (A) and *N. brasiliensis* FKB-3 and *C. elegans* FKB-3 (B). RMSD, the distance between the backbones of superimposed proteins. Total RMSD, the average RMSD for all molecules. The position of motifs analyzed by Prosite is indicated by bars. ^1^Hsp20 family, which includes a variety of small Hsps. ^2^FKBP-type peptidyl-prolyl cis-trans isomerase domain.
(TIF)

Figure S6 Hsp-gene expression levels of *N. brasiliensis* recovered from *rnu/rnu* rats (open columns) and *rnu*/+ rats (closed columns). At 21 days PI, as all worms had been expelled from *rnu*/+ rats, only data from *rnu/rnu* rats are shown. Gene expression levels are normalized to those of *Nb*-globin b. The levels in worms recovered from *rnu*/+ rats at 7 days PI are expressed as 1.0.
(TIF)
