# Supplementary figures and images for: Hsp12.6 Expression Is Inducible by Host Immunity in Adult Worms of the Parasitic Nematode Nippostrongylus brasiliensis
Source: PLoS One. 2011 Mar 23;6(3):e18141. doi: 10.1371/journal.pone.0018141 (PMC3063176; doi:10.1371/journal.pone.0018141)

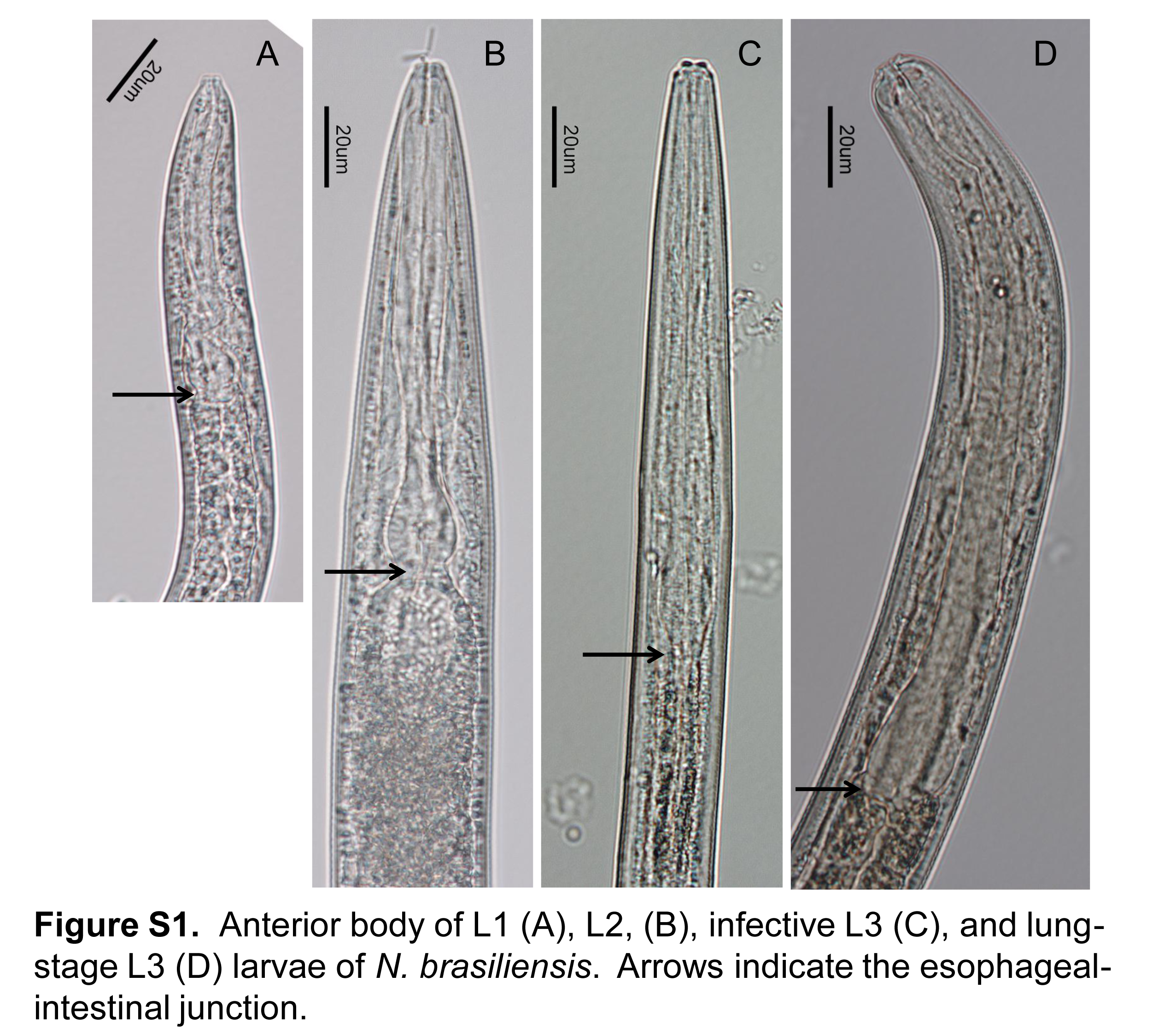

Supplement: Figure S1 — Anterior body of L1 (A), L2, (B), infective L3 (C), and lung-stage L3 (D) larvae of N. brasiliensis. Arrows indicate the esophageal-intestinal junction. (TIF) [file pone.0018141.s001.tif]

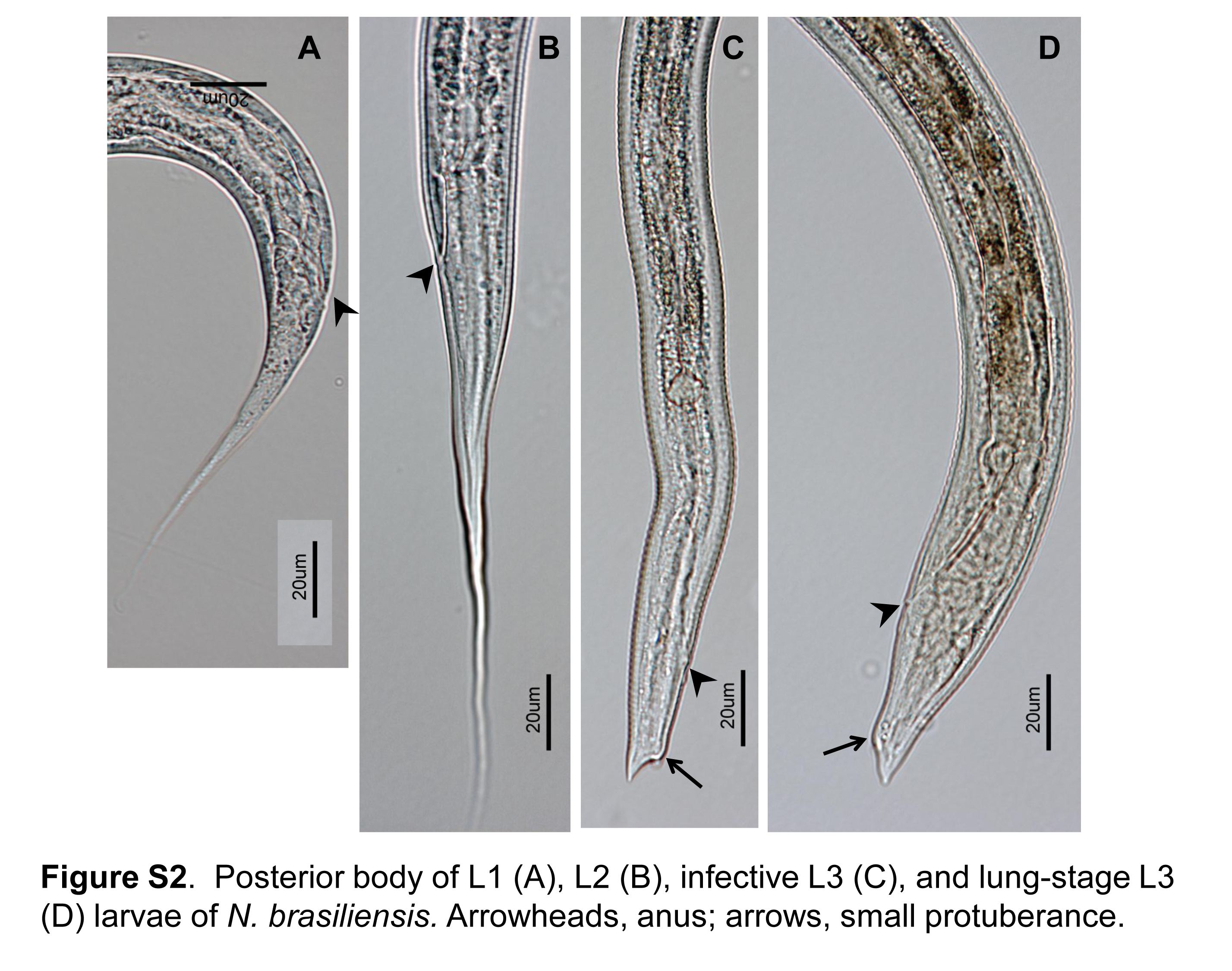

Supplement: Figure S2 — Posterior body of L1 (A), L2 (B), infective L3 (C), and lung-stage L3 (D) larvae of N. brasiliensis. Arrowheads, anus; arrows, small protuberance. (TIF) [file pone.0018141.s002.tif]

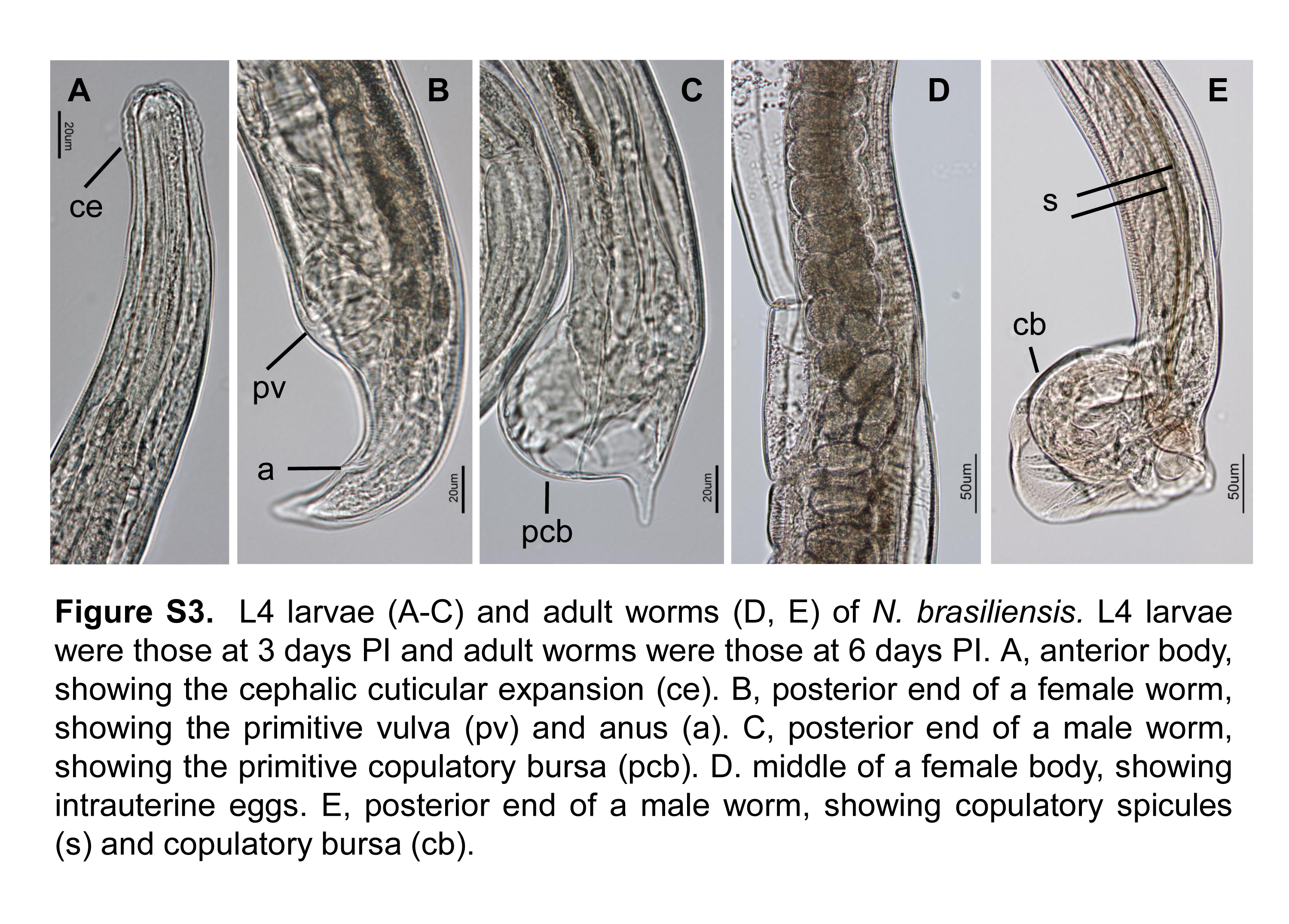

Supplement: Figure S3 — L4 larvae (A–C) and adult worms (D, E) of N. brasiliensis. L4 larvae were those at 3 days PI and adult worms were those at 6 days PI. A, anterior body, showing the cephalic cuticular expansion (ce). B, posterior end of a female worm, showing the primitive vulva (pv) and anus (a). C, posterior end of a male worm, showing the primitive copulatory bursa (pcb). D. middle of a female body, showing intrauterine eggs. E, posterior end of a male worm, showing copulatory spicules (s) and copulatory bursa (cb). (TIF) [file pone.0018141.s003.tif]

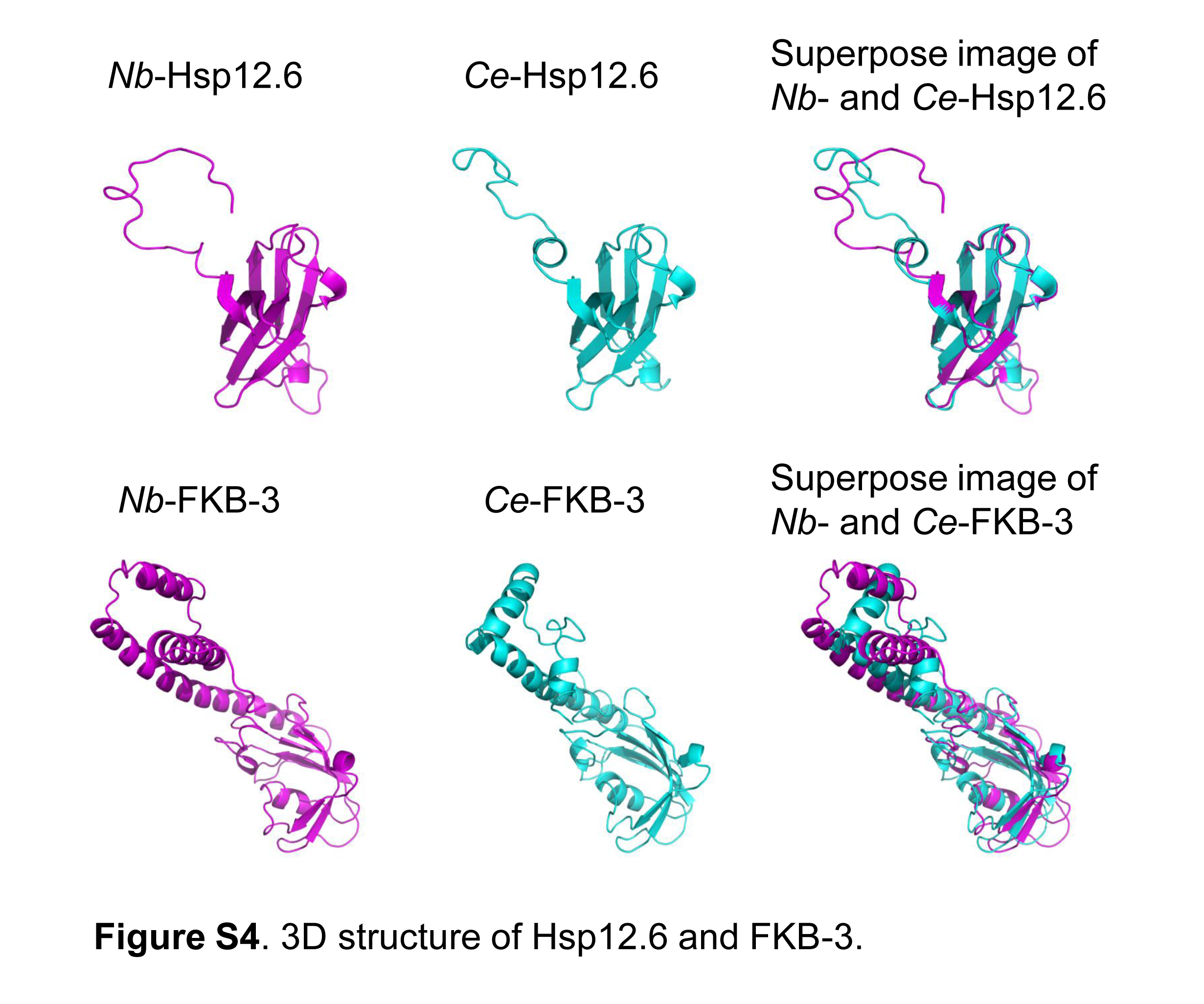

Supplement: Figure S4 — 3D structure of Hsp12.6 and FKB-3. (TIF) [file pone.0018141.s004.tif]

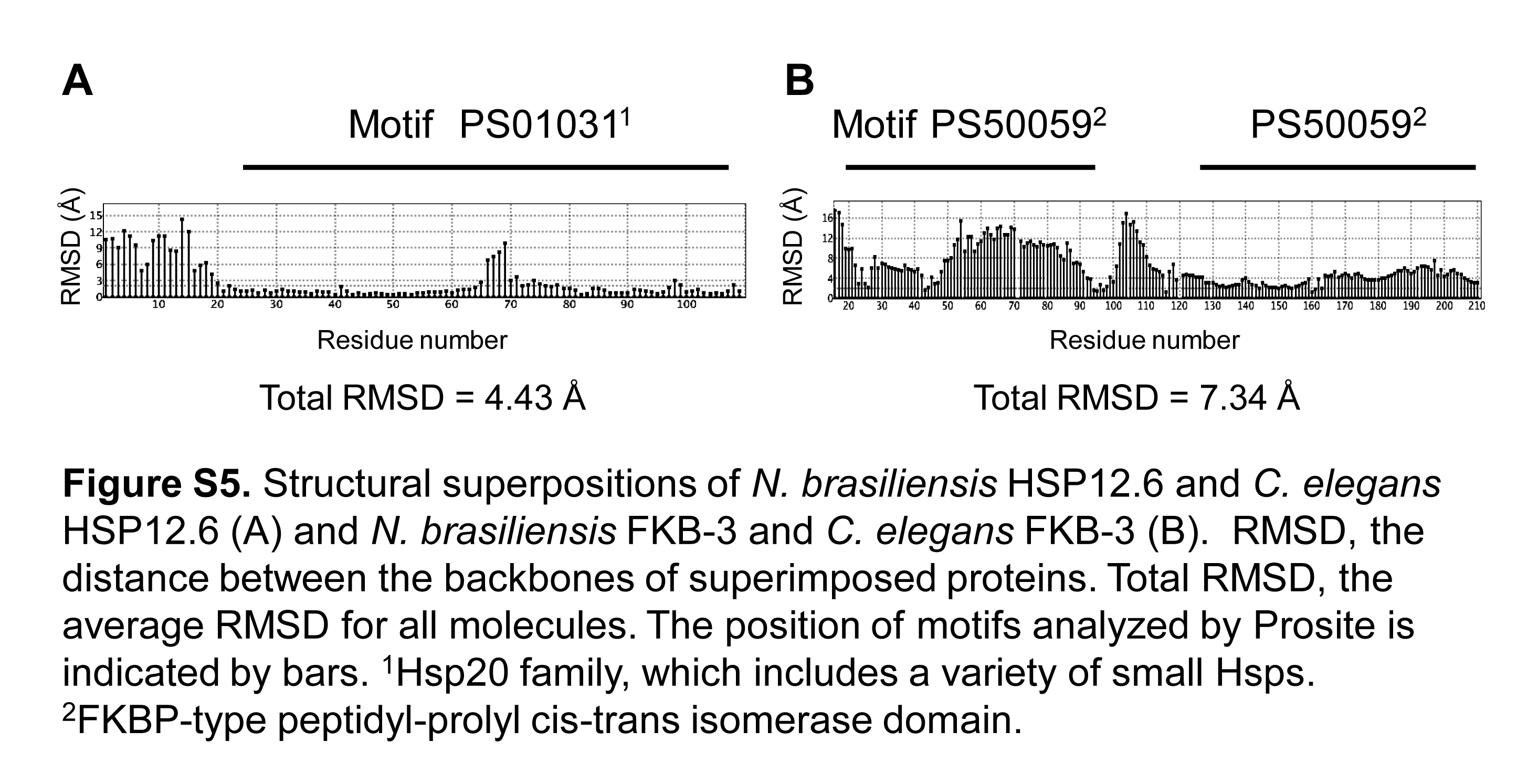

Supplement: Figure S5 — Structural superpositions of N. brasiliensis HSP12.6 and C. elegans HSP12.6 (A) and N. brasiliensis FKB-3 and C. elegans FKB-3 (B). RMSD, the distance between the backbones of superimposed proteins. Total RMSD, the average RMSD for all molecules. The position of motifs analyzed by Prosite is indicated by bars. 1Hsp20 family, which includes a variety of small Hsps. 2FKBP-type peptidyl-prolyl cis-trans isomerase domain. (TIF) [file pone.0018141.s005.tif]

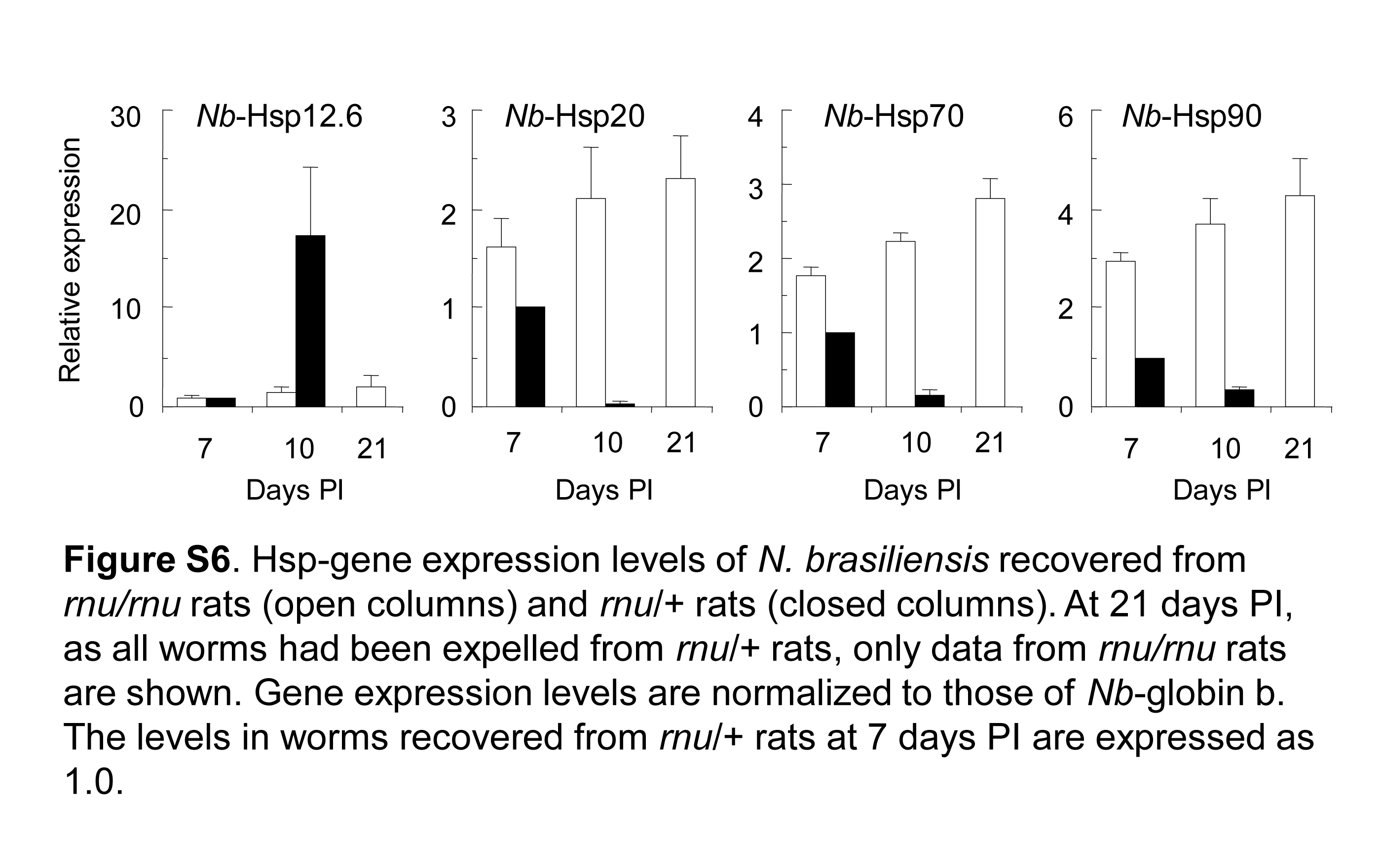

Supplement: Figure S6 — Hsp-gene expression levels of N. brasiliensis recovered from rnu/rnu rats (open columns) and rnu/+ rats (closed columns). At 21 days PI, as all worms had been expelled from rnu/+ rats, only data from rnu/rnu rats are shown. Gene expression levels are normalized to those of Nb-globin b. The levels in worms recovered from rnu/+ rats at 7 days PI are expressed as 1.0. (TIF) [file pone.0018141.s006.tif]
